# Crypsis via leg clustering: twig masquerading in a spider

**DOI:** 10.1098/rsos.150007

**Published:** 2015-03-04

**Authors:** Shichang Zhang, Kuei-Kai Mao, Po-Ting Lin, Chiu-Ju Ho, Wei Hung, Dakota Piorkowski, Chen-Pan Liao, I-Min Tso

**Affiliations:** 1 Department of Life Science, and, Tunghai University, Taichung 40704, Taiwan; 2 Center for Tropical Ecology and Biodiversity, Tunghai University, Taichung 40704, Taiwan

**Keywords:** camouflage, contour modification, twig-mimicking, crypsis, *Ariamnes cylindrogaster*

## Abstract

The role of background matching in camouflage has been extensively studied. However, contour modification has received far less attention, especially in twig-mimicking species. Here, we studied this deceptive strategy by revealing a special masquerade tactic, in which the animals protract and cluster their legs linearly in the same axis with their bodies when resting, using the spider *Ariamnes cylindrogaster* as a model. We used cardboard papers to construct dummies resembling spiders in appearance and colour. To differentiate the most important factors in the concealment effect, we manipulated body size (long or short abdomen) and resting postures (leg clustered or spread) of the dummies and recorded the responses of predators to different dummy types in the field. The results showed that dummies with clustered legs received significantly less attention from predators, regardless of the body length. Thus, we conclude that *A. cylindrogaster* relies on the resting posture rather than body size for predator avoidance. This study provides, to the best of our knowledge, empirical evidence for the first time that twig-mimicking species can achieve effective camouflage by contour modification.

## 2. Introduction

Avoiding predation is crucial for animals, and a variety of defensive strategies are adopted to cope with the pressures imposed by predators. The major strategy of predation avoidance is to decrease predators' detection and recognition by camouflage [1]. This strategy is normally achieved by deceptively incorporating background matching features, such as coloration and morphology, with which an animal can closely resemble the colour and the patterns of its background. Another way to achieve camouflage is contour modification (or disruptive coloration), in which an animal partly blends with the background by obscuring its contour and true form [2,3] to prevent predators from using edges and boundaries to visually recognize it [4].

Masquerading is one type of camouflage, which refers to organisms whose behaviour and/or appearance deceives observers into misidentifying the organism as an inanimate object found in the environment, and consequently, enhances the survival of the masquerader [5]. Inanimate objects masquerading organisms have been found to resemble include: twigs, leaves, stones and bird dropping [6,7]. Masquerading species can be found in a diverse array of taxa such as insects, fishes, sea anemones and birds [8,9].

Twig-mimicking is a common defensive strategy adopted by prey animals such as stick insects and caterpillars of some moth species [8]. By resembling twigs, these animals can potentially prevent themselves from being detected by predators, such as birds or predatory wasps [1,5]. While camouflage via background matching has been extensively investigated [10], predator defence through modification of body outlines and forms has received far less attention [11–13] and empirical support is scarce [14,15].

Many twig-mimicking insects share a characteristic feature, in which they rest in a cryptic posture with legs pressed against body and extended in parallel to the long axis of the body. In addition to protracting legs, some insects may rest with postures that make their head and antennae well concealed, creating a more linear appearance [16,17]. Such cryptic resting posture and the overall body coloration of the insects can potentially make them inconspicuous to predators, such as birds or predatory wasps, which mainly rely on visual cues to detect prey [17]. This phenomenon is also found in spider species of the genera *Miagrammopes* (Uloboridae) and *Ariamnes* (Theridiidae), which rest on a single horizontal thread during daytime, with legs extending forward and backward from the body ([18] and S. Zhang's 2012, personal observation). Although Eberhard [19] proposed that in some spiders, such as *Ulobrus diversus* (Uloboridae), crypsis occurs mostly through an extended leg posture rather than background colour matching, however, the contribution of this posture to masquerading success has not, to our knowledge, been tested experimentally. Additionally, how various camouflage modes interact to enhance survival of organisms has received very little investigation [20].

Here, we studied the role of contour modification in twig masquerading using the spider *Ariamnes cylindrogaster* (Araneae: Theridiidae) (see the electronic supplementary material, figure S1) as the model organism. Spiders of the genus *Ariamnes* are araneophagic nocturnal species characterized by a very elongated abdomen [21,22]. During the day, this spider will rest on a single horizontal silk thread with four legs clustered together extending forward and the other four backward [21,22]. Predatory wasps (e.g. mud-dauber wasps) have been considered the major predator of web-building spiders [23], and twig-mimicking insects often become the victim of these wasps [1]. Therefore, we hypothesized that *A. cylindrogaster* masquerades through a leg-clustering posture and an elongated body, so can decrease predation from predators, such as the predatory wasps. To test this hypothesis, we performed field experiments using dummy spider replicates, and video cameras to monitor predators' response to dummies with different body length and leg postures.

## 3. Material and methods

### 3.1 Construction of dummies

The use of dummy spiders allowed us to freely manipulate the posture and body length of spiders. To test the effects of abdomen length and leg posture on predator detection, four types of dummies were constructed: long abdomen with legs clustered (LC), long abdomen with legs spread (LS), short abdomen with legs clustered (SC) and short abdomen with legs spread (SS) (electronic supplementary material, figure S2). Dummies were not differentiated by sex during the construction process because males are morphologically similar to females in *A. cylindrogaster* [21,22]. In addition, we made the leg spread dummies to be bilaterally symmetric. We collected various commercially available cardboard papers to construct dummies. To determine which cardboard colour signal was most similar to that of the body of *A. cylindrogaster*, colour contrast of each cardboard paper when seen against the body colour of *A. cylindrogaster* was calculated. Colour contrast is the spectral difference between two objective areas and can only be detected by a visual system with at least two photoreceptor types [24]. To calculate colour contrast, we used the illumination spectrum (the spectrum of the light source), the object's reflectance spectrum and the spectral sensitivities of all photoreceptor types in insects' visual system [24]. We used the neuroethological model developed for honeybees (upon which intensive visual physiological studies have been conducted [25]) to calculate chromatic and colour contrasts of cardboards and spider body parts when viewed against each other because the predators recorded in our study site were wasps.

Reflectance spectra of cardboards were measured in the laboratory at Tunghai University by spectrometer (S4000, Ocean Optics, Inc., Dunedin, FL, USA). Those of *A. cylindrogaster* were obtained from four adult females collected from the study site in which the field experiments were conducted following the methods of Tso *et al*. [26]. We also measured the reflectance spectra of twigs collected from five different plants (*Prunus taiwaniana*, *Duranta erecta*, *Murraya paniculata*, *Tabernaemontana divaricate* and *Ehretia resinosa*) in the study site. Twigs of these plants might be the models that the spiders are mimicking. For each cardboard paper, spider and twig, we randomly selected six points to measure reflectance spectra and the tip of the probe was placed vertically 5 mm above the objects measured. Colour signals were generated by multiplying the surface reflectance function by the irradiance of the habitat [27]. The surface reflectance is the fraction of the light reflected from the surfaces of the cardboards, spiders or twigs. Based on the colour hexagon model [24,28,29], combined with calculated colour signals and the spectral sensitivity of honeybees, colour contrast values were calculated. In this analysis, we assumed a photoreceptor discrimination threshold value of 0.11 hexagon units. There are reports of honeybee colour discrimination thresholds as low as 0.04 hexagon units for differentially conditioned bees. However, we considered 0.11 to be appropriate to use because it accounts for the different sensitivities of the ultraviolet, blue and green photoreceptors of absolute conditioned or unconditioned bees [30]. Papers were chosen if the colour contrast value was smaller than or close to 0.11.

### 3.2 The field experiment

Field experiments were conducted in the Firefly Conservation Garden, Dakeng, Taichung City, Taiwan (120°45′58.0′′ E, 24°10′46.1′′ N) on 12–17 August 2012 and 4–12 May 2013. The study site was located on a trail winding through a secondary subtropical forest. The main tree species in the study site included the tung oil tree (*Aleurites fordii* and *Aleurites montana*) and formosa acacia (*Acacia confusa*). During late spring and summer, *A. cylindrogaster* are quite abundant in the study site. We used video cameras (Sony DCR-SR62 and 100) to record the responses of predatory insects to different types of dummies, and the recordings were made 8 h a day (from 08.00 to 16.00). Before recording, dummy spiders were attached to a thread of spider major ampullate silk (collected from the giant wood spider *Nephila pilipes*, which is also quite common in the study site) tied horizontally between two twigs at locations where *A. cylindrogaster* had been previously found. The silk thread was 1 m long and was placed about 1 m above the ground. Video cameras were placed 1–2 m away from the dummy spiders, depending on potentially interfering vegetation nearby. During the field experiments, around 20 video cameras were used each day. After the completion of field experiments, we viewed video footage noting events of predators flying around or contacting with dummy spiders. Among all the approaching insects, we only considered hymenopteran insects as potential predators, and we considered the predator had detected the dummy if it was flying closely around the dummy (within 5 cm distance) to inspect the dummy longer than 10 s or had directly touched the dummy after inspection. For each individual dummy used, the number of such events was totalled and divided by the number of monitoring hours to calculate the predator approach rate (number of predator approached per hour of monitoring).

### 3.3 Statistical analysis

We used one-tailed *t*-tests to compare colour contrast values of the papers used for constructing the dummies when seen against the body colour of *A. cylindrogaster* with the discrimination threshold values of 0.04 and 0.11. In addition, colour contrast values of spectrum of paper used to construct dummy, abdomen of the spider and potential twig model when seen against each from the eyes of honeybees were calculated. One-tailed *t*-tests were used to compare these values with the colour discrimination threshold values estimated for honeybees (0.04 and 0.11, respectively). A mixed-effect Poisson regression model was used to ascertain the effects of treatment, year and their interaction on predator approach rates. We dropped the independent variables sequentially from a full model to develop a reduced model. The explanatory variables included three fixed effects (dummy's abdomen form, dummy's leg arrangement and interaction; table 1) and one random intercept according to ‘year’. Video recording time (in hours) of each dummy sample was considered as the offset in the model. A Pearson *χ*^2^ goodness-of-fit test showed that this model reasonably fitted the data (*χ*^2^_145_=137.2, *p*=0.6649).

**Table 1.** Chromatic colour contrast values of various objects when seen against each other from the eyes of honeybees and results of one-tailed *t*-test comparing these values with two discrimination thresholds (0.04 and 0.11).

			HA: *μ*<0.04		HA: *μ*<0.11	
comparison	chromatic colour contrast (mean±s.e.m.)	d.f.	*T*	*P*	*T*	*P*
spider versus dummy	0.025±0.008	3	−1.830	0.082	−10.500	0.0009
spider versus twig	0.071±0.013	3	2.433	0.953	−3.105	0.027
twig versus dummy	0.053±0.015	4	0.870	0.793	−3.717	0.0103

## 4. Results

**Figure 1.**
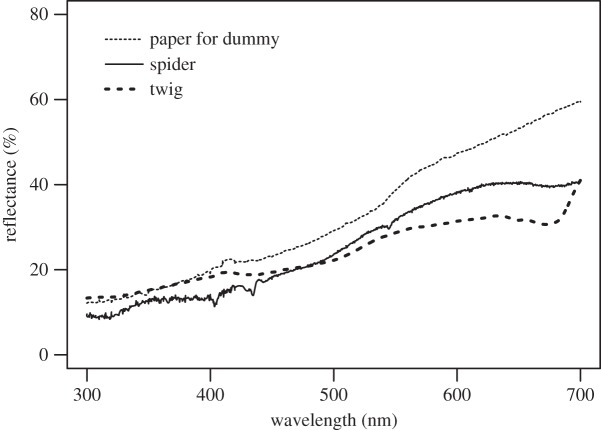
Reflectance spectrum of body colour of female *Ariamnes cylidrogaster*, paper used to construct dummies and potential twig model.

The reflectance spectra of the body colour of *A. cylindrogaster*, cardboard paper used to construct dummies and potential twig models are given in figure 1. The chromatic colour contrast values of various objects when seen against each other are given in table 1. Results of one-tailed *t*-tests showed that the value of papers used to construct dummies was significantly smaller than the 0.11 discrimination threshold (*t*_3_=−10.5, *p*=0.0009). This result indicates that from the eyes of hymenopteran insects the colour of the dummies and *A. cylindrogaster* are indistinguishable (table 1). This conclusion is valid even if a 0.04 discrimination threshold value is assumed because our chromatic colour contrast values do not significantly differ from such value (table 1). Similarly, the results of *t*-tests also showed that the colour contrast of dummies, spiders and twigs when seen against each other were not significantly higher than the discrimination threshold of 0.04 or 0.11.

**Figure 2.**
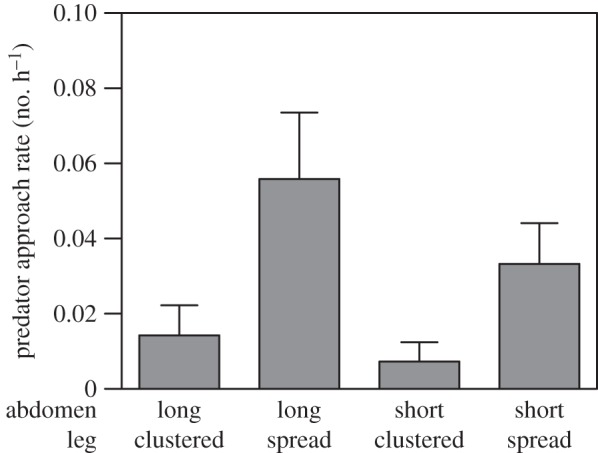
Mean (±s.e.) predator approach rates of four types of dummies used in the field experiments.

In 2012, a total of 988 h of video monitoring was obtained and in 2013, 295 h was obtained. Result of a preliminary analysis using the Poisson mixed-effect full model showed that the effect of year was not significant, so we dropped this factor and analysed the predator approach data with a reduced model. Pooled from 2 years of field experiments the sample sizes of the LC, LS, SC and SS treatment groups were 35, 37, 35 and 43, respectively. Results of the reduced model showed that there was no interaction between the abdomen length and leg posture (table 2). Results of the reduced model also showed that the effect of abdomen length on predator approach rates was not significant. However, the leg posture of the dummies had a significant effect (table 2). Dummies with legs clustered were significantly less likely to be approached by hymenopteran predators (mostly members of the family Sphecidae), regardless of having a short or long abdomen (figure 2).

**Table 2.** Results of a mixed-effects Poisson regression model comparing the predator approach events of dummies with different abdomen form and leg posture. (The *β* of the long abdomen and leg clustered dummy group was arbitrarily designated as 0 to facilitate comparison of probabilities of different events. The ratio between probabilities of two certain events was *e*^*β*^.)

coefficient of fixed effect	estimate of *β*	s.e.	*z*	*p*-value
intercept	−3.915	0.257	−15.223	<0.0001
abdomen (short–long)	−0.466	0.514	−0.905	0.365
leg (spread–clustered)	1.173	0.514	2.280	0.023
interaction	0.103	1.029	0.100	0.920

## 5. Discussion

We studied the effectiveness of twig masquerading in decreasing predator's detection using *A. cylindrogaster* as a model organism. Here, we demonstrate, to the best of our knowledge, for the first time that twig masquerading achieved by modification of body outlines and forms can effectively divert attention of predators from prey. Obscuring contours via disruptive coloration has been intensively reported [5,14] and such phenomenon has been found in animal taxa such as mammals [4,31], fishes [31,32], isopods [4] and cephalopods [33]. Strictly, leg-clustering in our case is masquerading rather than disruptive coloration, because body contours of the spiders are still conspicuous but are modified, so that they are not recognized as a prey item (figure 2). In addition, *A. cylindrogaster* does not have direct contact with the background, while in most animals with disruptive coloration they usually have direct contact with the background [4]. Concealing body contours by clustering legs against the body have been rarely reported and most examples are found in insects [17] and spiders [18,19] with elongated bodies. However, in this study, we found that the length of the body is not as important as the leg posture for effective masquerading, as leg-clustered dummies with long bodies did not differ significantly to short bodies in predator approach rates. In many twig-mimicking species, the length of the body varies considerably, which may be due to different degrees of nutritional intake or a reflection of evolutionary or ecological constraints. Skelhorn *et al*. [8] demonstrated that matching an inanimate model object in size is not necessary for a successful masquerading, although appearance similarity plus size matching could maximize the protection effect. In addition, it has been reported that hymenopteran insects, such as bumblebees (*Bombus terrestris*) innately preferred objects that were bilaterally symmetric [34,35]. Thus, it is unknown whether and how much such preference has contributed to the high attraction of the leg spread dummies, which are visually bilaterally symmetric. We suggest future studies may investigate how much the linear shape of the twig-mimicking spider mutes the visual stimulus of bilateral symmetry in the eyes of predatory wasps.

Many masquerading organisms choose habitats with background coloration similar to that of their own body coloration, as visual elements of the habitat can effectively deceive the predators through background matching [4]. For example, the overall colour and appearance of some spider species from genera such as *Poltys* and *Dolophenes* (family Araneidae) and *Tetragnatha* (family Tetragnathidae) provide excellent camouflage when they are resting on dead twigs (S. Zhang's 2012, personal observation). However, there are some other masqueraders with body colours exhibiting high contrast with the background. For example, bolas spiders (*Mastophora* spp., Araneidae) and crab spiders (*Cyrtarachne* spp., Thomisidae) exhibit a conspicuous bird-dropping appearance that sharply contrasts in coloration with their resting microhabitats [36–38]. Similarly, some species with leg-clustering postures also have body colours that contrast conspicuously with the background, such as the silver-bodied kleptoparasitic spider *Argyrodes fissifrons* (Theridiidae) [39], and the red-bodied social spider *Philoponella alata* (Uloboridae) [40]. Nevertheless, these conspicuous body colours may be involved in prey attraction rather than predator avoidance [39]. Evidently, the coexistence of low and high contrast body colours in these spiders represents a trade-off between prey and predator attraction. In *A. cylindrogaster*, mimicking a twig by clustering their legs may further enhance the effect of crypsis.

In our study, *A. cylindrogaster* usually rests on a single silk thread far away from the twigs where the thread was anchored. It was always found in the context where the general colour of the surroundings matches its body colour. In many other spider species from genera such as *Miagrammopes* and *Ulobrus* (family Uloboridae) that potentially adopt twig masquerading, they may have also taken advantage of background matching. According to our observations, these uloborid spiders have either grey or green colour types and inhabit different environments. Although the role body coloration might play in crypsis remains unknown, there is little doubt that effective background matching will decrease the contrast between the body and the background, hence strengthening the effect of twig masquerading [20]. Future studies should examine the roles of body coloration in concealment of these species and investigate whether these twig masqueraders are able to enhance camouflage by changing their body colours according to the colours of their surroundings or selecting microhabitats that best match their coloration [41].

In this study, we empirically demonstrate a special masquerade tactic in spiders, which provides a new perspective in understanding the role of contour modification in the camouflage of animals. By manipulating the body length and leg postures of dummy spiders we were able to show that spiders can well conceal themselves from predators' attention by behaviourally adjusting body postures to create false contours mimicking a twig. Understanding how background matching and contour modification interacts to achieve deceiving would allow us to predict how selection pressures shape the appearance of these leg-clustering animals. We suggest that future studies follow the approach used in this study to investigate how morphological and behavioural anti-predator adaptations interact to enhance the survival of organisms.

## Data accessibility

The supporting data are made available as electronic supplementary material.

## References

[1] Ruxton GD, Sherratt TN, Speed MP 2004 Avoiding attack: the evolutionary ecology of crypsis, warning signals and mimicry. New York, NY: Oxford University Press.

[2] Cott H 1940 Adaptive coloration in animals. London, UK: Methuen.

[3] Edmunds M 1974 Defence in animals. Harlow, UK: Longman.

[4] Merilaita S 1998 Crypsis through disruptive coloration in an isopod. Proc. R. Soc. Lond. B 265, 1059–1064. (doi:10.1098/rspb.1998.0399)

[5] Skelhorn J, Rowland HM, Ruxton GD 2010 The evolution and ecology of masquerade. Biol. J. Linn. Soc. 99, 1–8. (doi:10.1111/j.1095-8312.2009.01347.x)

[6] Skelhorn J, Ruxton GD 2011 Mimicking multiple models: polyphenetic masqueraders gain additional benefits from crypsis. Behav. Ecol. 22, 60–65. (doi:10.1093/beheco/arq166)

[7] Endler JA 1981 An overview of the relationships between mimicry and crypsis. Biol. J. Linn. Soc. 16, 25–31. (doi:10.1111/j.1095-8312.1981.tb01840.x)

[8] Skelhorn J, Rowland HM, Speed MP, Ruxton GD 2010 Masquerade: camouflage without crypsis. Science 327, 51 (doi:10.1126/science.1181931) 20044568 10.1126/science.1181931

[9] Brooker RM, Munday PL, Jones GP 2011 Coral obligate filefish masquerades as branching coral. Coral Reefs 30, 803–803. (doi:10.1007/s00338-011-0779-6)

[10] Skelhorn J, Rowland HM, Delf J, Speed MP, Ruxton GD 2011 Density-dependent predation influences the evolution and behavior of masquerading prey. Proc. Natl Acad. Sci. USA 108, 6532–6536. (doi:10.1073/pnas.1014629108) 21464318 10.1073/pnas.1014629108 PMC3081003

[11] Stevens M, Winney IS, Cantor A, Graham J 2009 Outline and surface disruption in animal camouflage. Proc. R. Soc. B 276, 781–786. (doi:10.1098/rspb.2008.1450) 10.1098/rspb.2008.1450 PMC2660950 19019788

[12] Stevens M, Cuthill IC 2006 Disruptive coloration, crypsis and edge detection in early visual processing. Proc. R. Soc. B 273, 2141–2147. (doi:10.1098/rspb.2006.3556) 10.1098/rspb.2006.3556 PMC1635512 16901833

[13] Fraser S, Callahan A, Klassen D, Sherratt TN 2007 Empirical tests of the role of disruptive coloration in reducing detectability. Proc. R. Soc. B 274, 1325–1331. (doi:10.1098/rspb.2007.0153) 10.1098/rspb.2007.0153 PMC2176178 17360282

[14] Cuthill IC, Stevens M, Sheppard J, Maddocks T, Parraga CA, Troscianko TS 2005 Disruptive coloration and background pattern matching. Nature 434, 72–74. (doi:10.1038/nature03312) 15744301 10.1038/nature03312

[15] Schaefer HM, Stobbe N 2006 Disruptive coloration provides camouflage independent of background matching. Proc. R. Soc. B 273, 2427–2432. (doi:10.1098/rspb.2006.3615) 10.1098/rspb.2006.3615 PMC1634905 16959631

[16] Opell BD, Eberhard WG 1984 Resting postures of orb-weaving uloborid spiders (Araneae, Uloboridae). J. Arachnol. 11, 297–307. See http://www.jstor.org/stable/3705046

[17] Robinson MH 1969 The defensive behaviour of some orthopteroid insects from Panama. Trans. R. Ent. Soc. London. 121, 281–303. (doi:10.1111/j.1365-2311.1969.tb00521.x)

[18] Lubin Y, Eberhard W, Montgomery G 1978 Webs of *Miagrammopes* (Araneae: Uloboridae) in the neotropics. Psyche 85, 1–23. (doi:10.1155/1978/72579)

[19] Eberhard WG 1973 Stabilimenta on the webs of *Uloborus diversus* (Araneae: Uloboridae) and other spiders. J. Zool. 171, 367–384. (doi:10.1111/j.1469-7998.1973.tb05345.x)

[20] Merilaita S, Stevens M 2011 Crypsis through background matching. In Animal camouflage: mechanisms and function (eds Stevens M, Merilaita S), pp. 17–33. Cambridge, UK: Cambridge University Press.

[21] Gillespie RG, Rivera MAJ 2007 Free-living spiders of the genus *Ariamnes* (Araneae, Theridiidae) in Hawaii. J. Arachnol. 35, 11–37. (doi:10.1636/h04-05.1)

[22] Koh TH, Li DQ 2003 State-dependent prey type preferences of a kleptoparasitic spider *Argyrodes flavescens* (Araneae : Theridiidae). J. Zool. 260, 227–233. (doi:10.1017/s0952836903003674)

[23] Blackledge TA, Coddington JA, Gillespie RG 2003 Are three-dimensional spider webs defensive adaptations? Ecol. Lett. 6, 13–18. (doi:10.1046/j.1461-0248.2003.00384.x)

[24] Chittka L 1992 The colour hexagon: a chromaticity diagram based on photoreceptor excitations as a generalized representation of colour opponency. J. Comp. Physiol. A 170, 533–543. (doi:10.1007/BF00199331)

[25] Briscoe AD, Chittka L 2001 The evolution of color vision in insects. Annu. Rev. Entomol. 46, 471–510. (doi:10.1146/annurev.ento.46.1.471) 11112177 10.1146/annurev.ento.46.1.471

[26] Tso IM, Lin CW, Yang EC 2004 Colourful orb-weaving spiders, *Nephila pilipes*, through a bee's eyes. J. Exp. Biol. 207, 2631–2637. (doi:10.1242/jeb.01068) 15201295 10.1242/jeb.01068

[27] Wandell BA 1995 Foundations of vision. Sunderland, MA: Sinauer Associates.

[28] Chittka L 1996 Optimal sets of color receptors and color opponent systems for coding of natural objects in insect vision. J. Theor. Biol. 181, 179–196. (doi:10.1006/jtbi.1996.0124)

[29] Chittka L 2001 Camouflage of predatory crab spiders on flowers and the colour perception of bees (Aranida: Thomisidae/Hymenoptera: Apidae). Entomol. Gen. 25, 181–187. (doi:10.1127/entom.gen/25/2001/181)

[30] Dyer AG, Chittka L 2004 Biological significance of distinguishing between similar colours in spectrally variable illumination: bumblebees (*Bombus terrestris*) as a case study. J. Comp. Physiol. A 190, 105–114. (doi:10.1007/s00359-003-0475-2) 10.1007/s00359-003-0475-2 14652688

[31] Stoner CJ, Caro TM, Graham CM 2003 Ecological and behavioral correlates of coloration in artiodactyls: systematic analyses of conventional hypotheses. Behav. Ecol. 14, 823–840. (doi:10.1093/beheco/arg072)

[32] Kelman EJ, Tiptus P, Osorio D 2006 Juvenile plaice (*Pleuronectes platessa*) produce camouflage by flexibly combining two separate patterns. J. Exp. Biol. 209, 3288–3292. (doi:10.1242/jeb.02380) 16916964 10.1242/jeb.02380

[33] Kelman EJ, Baddeley RJ, Shohet AJ, Osorio D. 2007 Perception of visual texture and the expression of disruptive camouflage by the cuttlefish, *Sepia officinalis*. Proc. R. Soc. 274, 1369–1375. (doi:10.1098/rspb.2007.0240) 10.1098/rspb.2007.0240 PMC2176201 17389219

[34] Rodríguez I, Gumbert A, Hempel de Ibarra N, Kunze J, Giurfa M 2004 Symmetry is in the eye of the ‘beeholder’: innate preference for bilateral symmetry in flower-naïve bumblebees. Naturwissenschaften 91, 374–377. (doi:10.1007/s00114-004-0537-5) 15278213 10.1007/s00114-004-0537-5

[35] Giurfa M, Eichmann B, Menzel R 1996 Symmetry perception in an insect. Nature 382, 458–461. (doi:10.1038/382458a0) 18610516 10.1038/382458a0

[36] Eberhard WG 1980 The natural history and behavior of the bolas spider, *Mastophora dizzydeani* sp. n. (Araneae). Psyche 87, 143–170. (doi:10.1155/1980/81062)

[37] Yeargan KV 1994 Biology of bolas spiders. Annu. Rev. Entomol. 39, 81–99. (doi:10.1146/annurev.ento.39.1.81)

[38] Cartan CK, Miyashita T 2000 Extraordinary web and silk properties of *Cyrtarachne* (Araneae, Araneidae): a possible link between orb-webs and bolas. Biol. J. Linn. Soc. 71, 219–235. (doi:10.1111/j.1095-8312.2000.tb01255.x)

[39] Peng P, Blamires SJ, Agnarsson I, Lin HC, Tso IM 2013 A color-mediated mutualism between two arthropod predators. Curr. Biol. 23, 172–176. (doi:10.1016/j.cub.2012.11.057) 23260470 10.1016/j.cub.2012.11.057

[40] Lin Y, Li S 2008 Description on a new *Philoponella* species (Araneae, Uloboridae), the first record of social spiders from China. Acta. Zootaxon. Sini. 33, 260–263

[41] Théry M, Casas J 2009 The multiple disguises of spiders: web colour and decorations, body colour and movement. Phil. Trans. R. Soc. B 364, 471–480. (doi:10.1098/rstb.2008.0212) 18990672 10.1098/rstb.2008.0212 PMC2674075

